# Supplementary material for: A multi-ancestry polygenic risk score for body mass index predicts longitudinal weight change
Source: Genome Med. 2026 Aug 5;18:130. doi: 10.1186/s13073-026-01741-8 (PMC13543510; doi:10.1186/s13073-026-01741-8)
Supplement: Supplementary file 1 [file 13073_2026_1741_MOESM2_ESM.pdf]

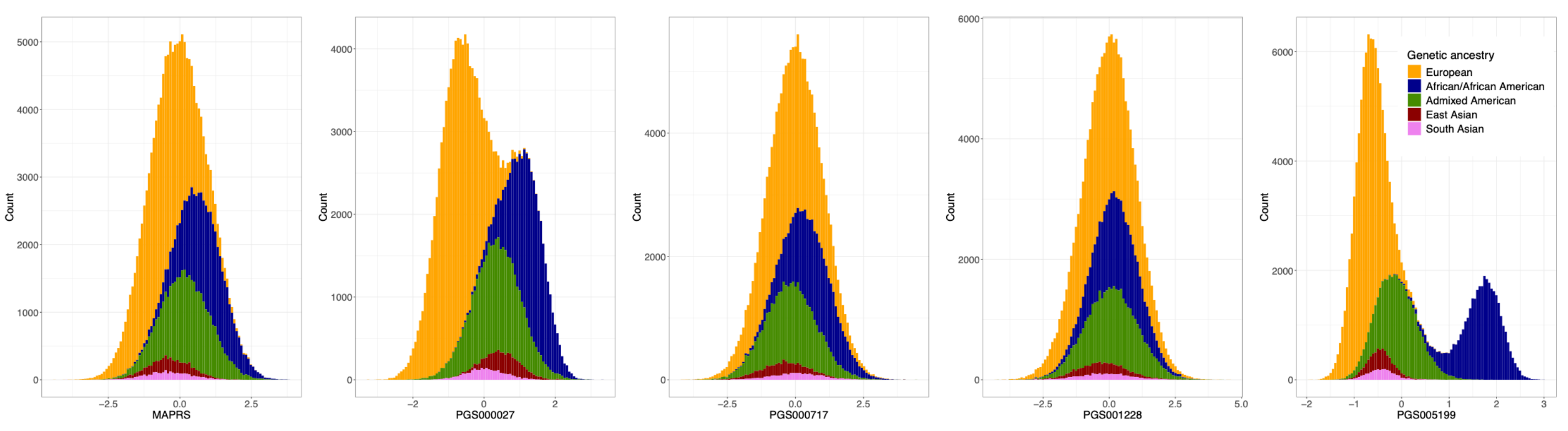

Fig. S1. Distribution of individual-level polygenic risk scores in the AoU test dataset. Histograms are stratified by genetic ancestry. PGS005199 exhibits substantial distributional differences across genetic ancestry groups.

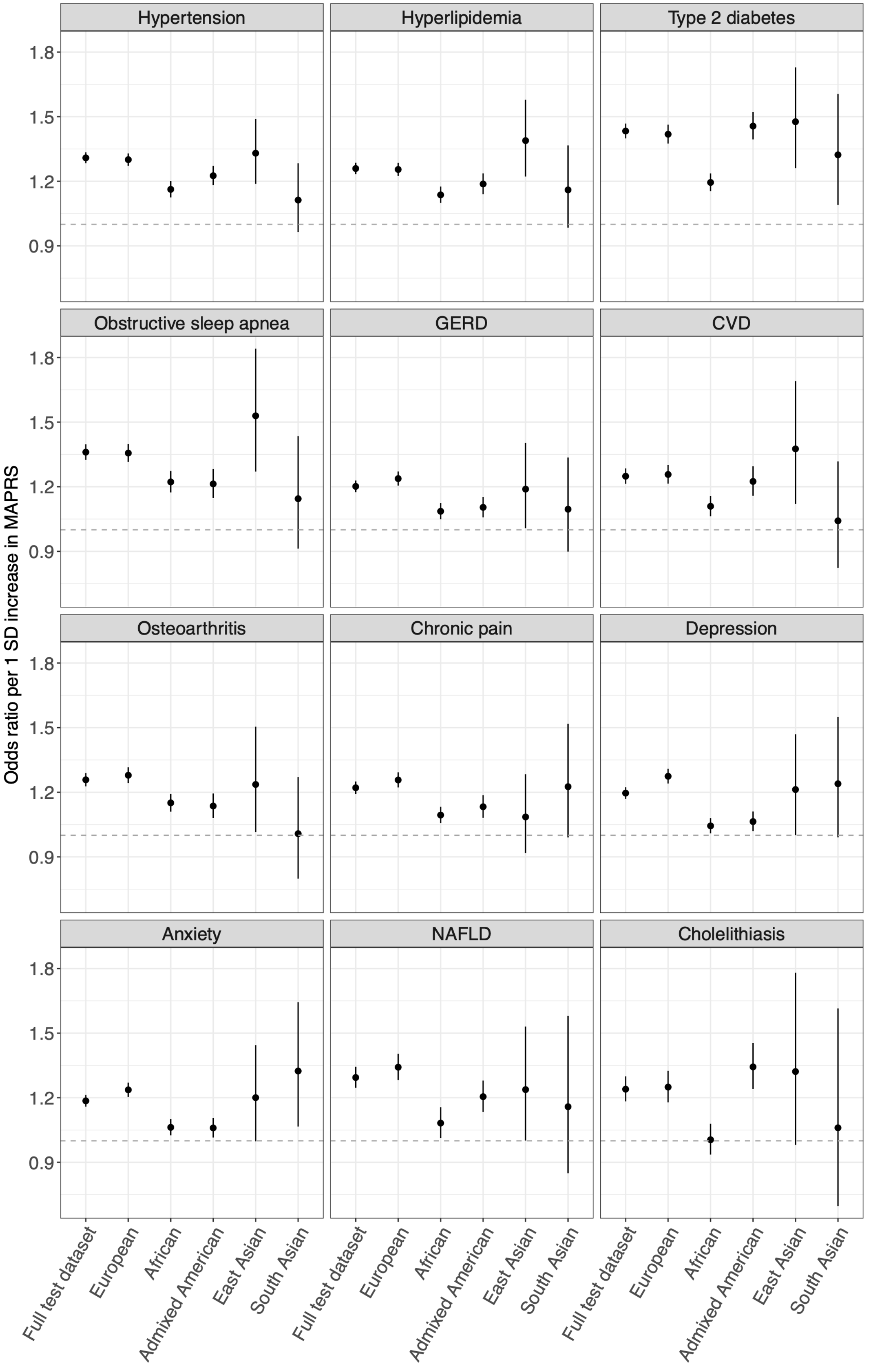

Fig. S2. Associations between the MAPRS and 12 clinical risk factors. Each dot represents the odds ratio for having the indicated clinical risk factor per one standard deviation increase in the MAPRS, estimated in the overall AoU test dataset and separately within each genetic ancestry group. Error bars indicate the 95% confidence intervals.

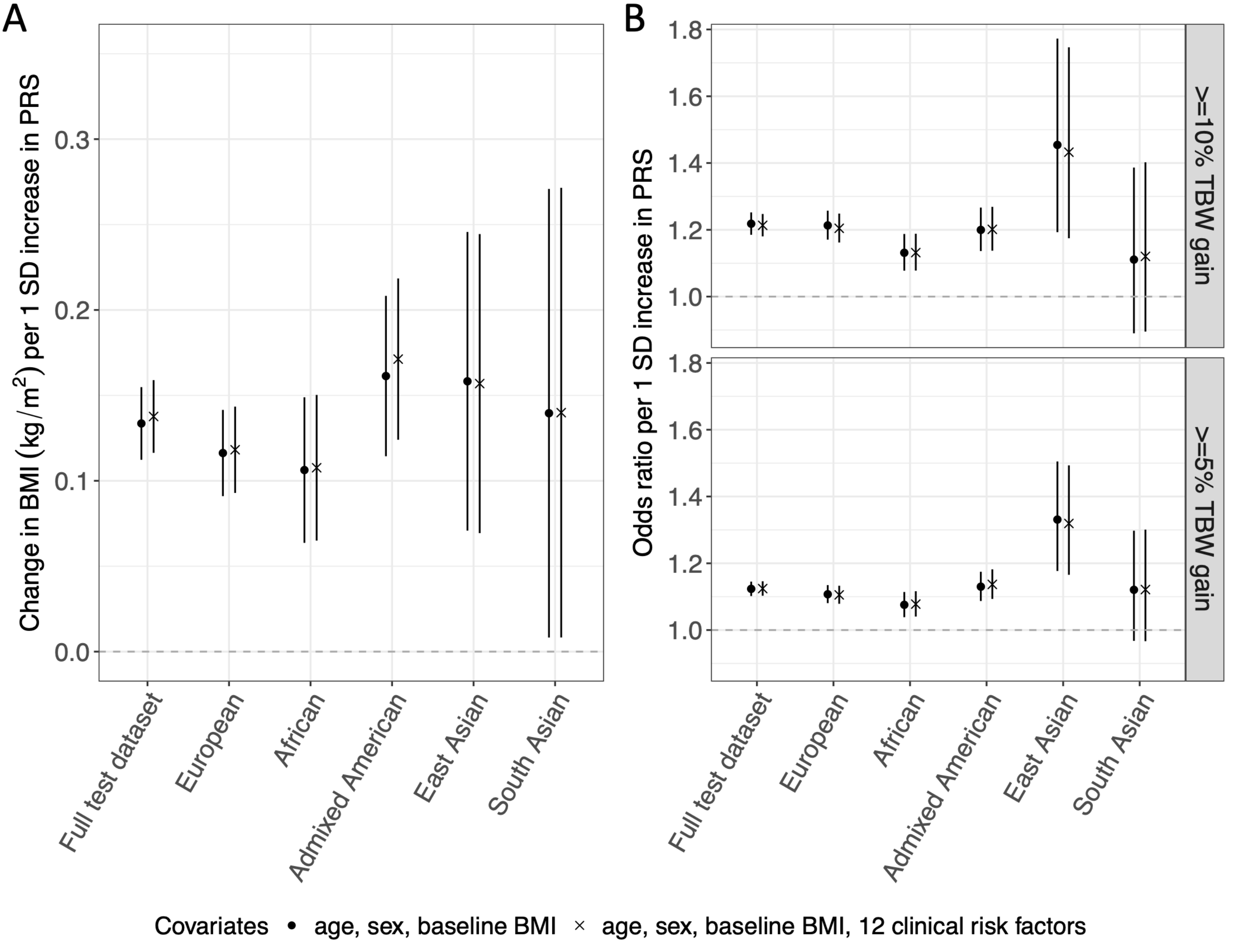

Fig. S3. Association between the MAPRS ($5x10^{-8}$) and weight change outcomes in the AoU test dataset. (A) The association between the MAPRS ($5x10^{-8}$) and change in BMI was assessed using linear regression. (B) Associations between the MAPRS ($5x10^{-8}$) and ≥10% or ≥5% TBW gain were assessed using logistic regression. Each dot represents the estimated effect size per one standard deviation increase in the MAPRS ($5x10^{-8}$), shown for the overall AoU test dataset and separately within each genetic ancestry group. Error bars indicate the 95% confidence intervals. All associations remained highly consistent after adjustment for clinical risk factors.

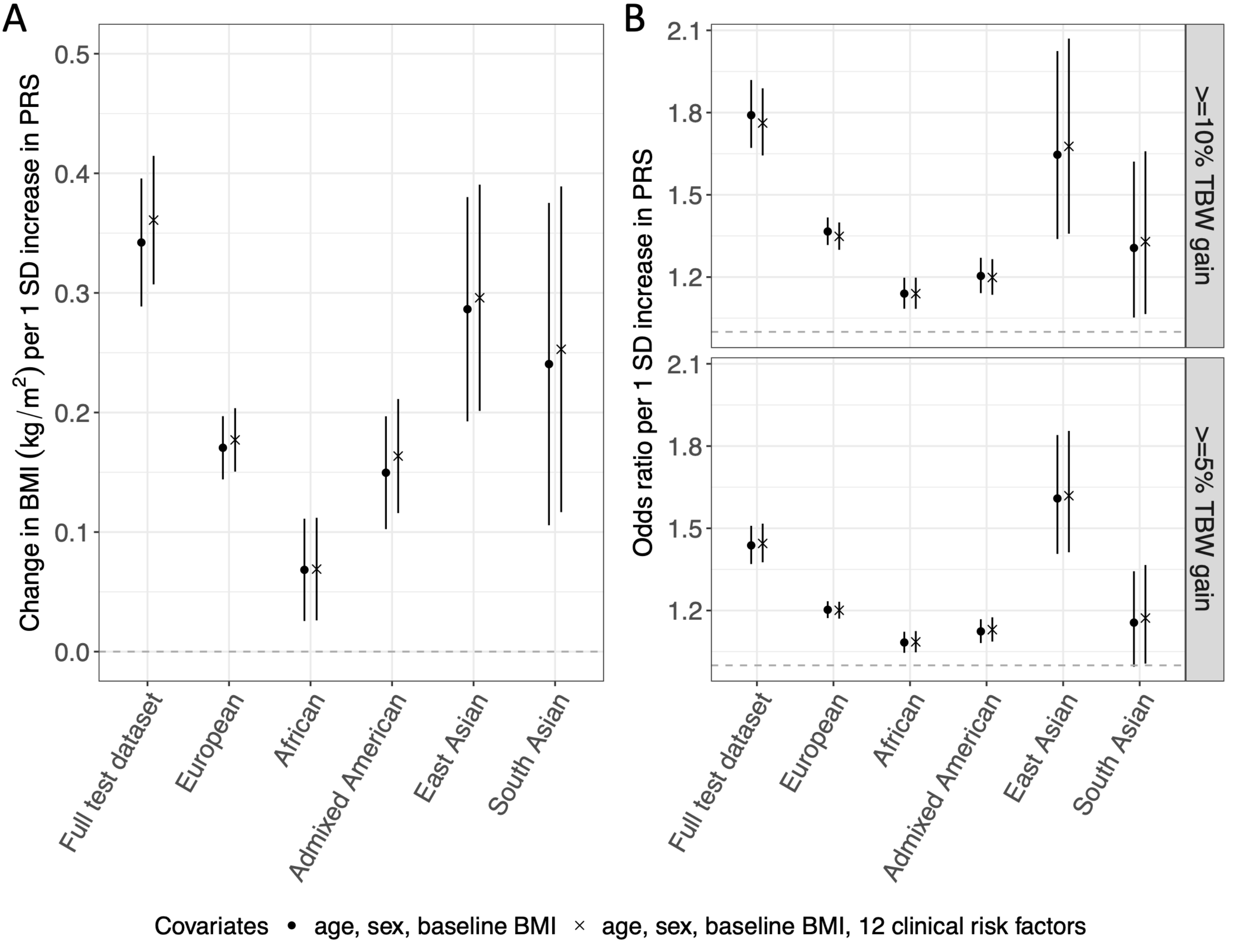

Fig. S4. Association between PGS005199 and weight change outcomes in the AoU test dataset. (A) The association between PGS005199 and change in BMI was assessed using linear regression. (B) Associations between PGS005199 and ≥10% or ≥5% TBW gain were assessed using logistic regression. Each dot represents the estimated effect size per one standard deviation increase in PGS005199, shown for the overall AoU test dataset and separately within each genetic ancestry group. Error bars indicate the 95% confidence intervals. All associations remained highly consistent after adjustment for clinical risk factors. Notably, the magnitude of the association may be stronger in the full test dataset than in any ancestry group for change in BMI and ≥10% TBW gain, suggesting a potential risk of confounding due to population stratification.
